# Quantification of myocardial perfusion with spiral pulse sequences

**DOI:** 10.1186/1532-429X-15-S1-E12

**Published:** 2013-01-30

**Authors:** Yang Yang, Sujith Kuruvilla, Craig H Meyer, Christopher M Kramer, Michael Salerno

**Affiliations:** 1 Biomedical Engineering, University of Virginia, Charlottesville, VA, USA; 2 Radiology, University of Virginia, Charlottesville, VA, USA; 3 Medicine, University of Virginia, Charlottesville, VA, USA

## Background

The quest to quantify myocardial perfusion has been largely motivated by the desire to obtain quantitative, observer-independent, and reproducible measures of myocardial perfusion. Spiral pulse sequences have multiple advantages for myocardial perfusion imaging including high acquisition efficiency, high signal to noise (SNR) and robustness to motion. We have developed a spiral pulse sequence with an integrated single-shot arterial input function (AIF) capable of performing absolute quantification of myocardial perfusion.

## Methods

**Figure 1:**
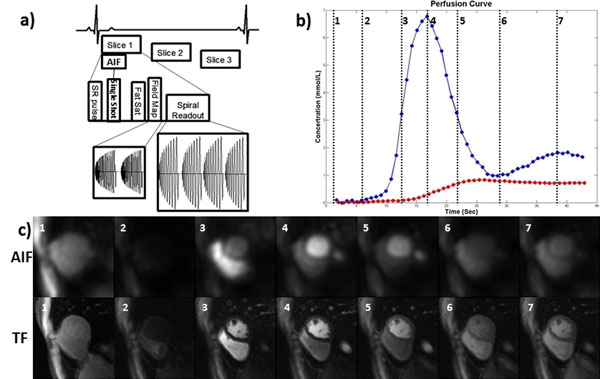
Schematic of the spiral absolute quantification sequence (a), tissue function and AIF tracer concentration curves (b) and the perfusion images at multiple time points(c).

Our previously optimized spiral pulse sequence was modified to additionally acquire proton density (PD) and arterial input function (AIF) images for quantification. The PD images were collected in the first 2 heart beats without a saturation pulse using a 10° flip angle (FA). The AIF was acquired during the saturation recovery time (SRT) of the first myocardial perfusion image (Figure. 1a), thus adding no additional imaging time to the sequence. Perfusion images were acquired on a 1.5T Siemens Scanner during injection of 0.1 mmol/kg of Gd-DTPA. Sequence parameters included: TE 1.0 ms, TR 9 ms, SRT 80 ms FA 35°, 3 slices with FOV 320 mm^2^, in-plane resolution 1.97 mm. AIF images were acquired with a single-shot spiral acquisition using a 90° FA with the following parameters: in-plane resolution 6.95 mm, SRT 20 ms. Imaging was performed in 7 human subjects. Perfusion images were normalized by the PD images and Bloch simulation was used to convert to absolute concentration-time curves prior to Fermi-function deconvolution. Quantification of perfusion was performed in MATLAB.

## Results

**Figure 2:**
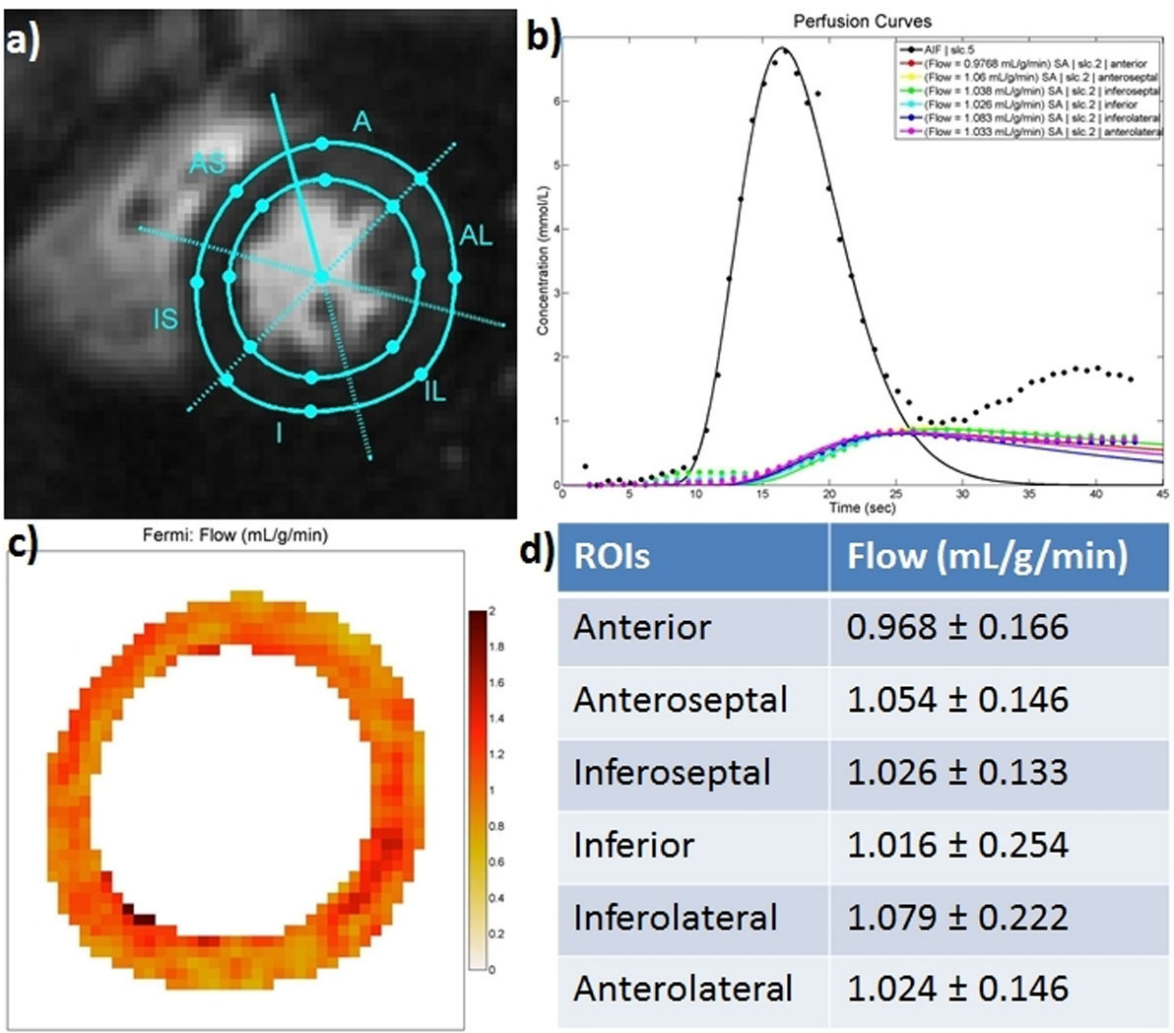
Segment ROIs (a), fitted perfusion curves (b), pixel-based flow map (c) and mean and standard deviation of the flow from different ROIs (d) from a normal healthy subject

Figure 1 shows the schematic of the sequence (a), tissue function (TF) and AIF tracer concentration curves (b) and TF and AIF images at the time points indicated on the time-concentration curves (c). The first time point shows the PD images. The images and concentration curves demonstrate high SNR. Figure 2 shows the segment ROIs (a), fitted perfusion curves (b), pixel-based flow map (c) and mean and standard deviation of the flow from different ROIs (d) from a normal subject. The mean absolute blood flow was 1.027±0.189 ml/g/min, which is very close to 1 ml/g/min expected for a healthy volunteer. The perfusion values are uniform across all of the ROIs.

## Conclusions

We demonstrated the successful application of a spiral-based pulse sequence for absolute quantification of perfusion in healthy volunteers. Preliminary studies using this technique appear promising but will require further validation in patients at rest and during adenosine stress.

## Funding

This work was supported by grants from AHA 10SDG2650038 and NIH K23 HL112910-01

